# Unlucky for some? Bleeding associated with factor XIII deficiency during extracorporeal membrane oxygenation

**DOI:** 10.1016/j.rpth.2026.103363

**Published:** 2026-02-06

**Authors:** Andrew J. Doyle

**Affiliations:** Haemostasis and Thrombosis Centre, Guy’s & St Thomas’ National Health Service Foundation Trust, London, UK

Both bleeding and thrombotic events are common complications during extracorporeal membrane oxygenation (ECMO). Bleeding is associated with an increased risk of death during ECMO, with large multicenter international registry data describing hospital-related mortality with bleeding at 40% [1]. Multiple hemostatic changes having been recognized in the development of bleeding during ECMO including thrombocytopenia and platelet dysfunction, hypofibrinogenemia, acquired von Willebrand dysfunction, and overdosing of anticoagulation [2].

In this issue, Kornfehl et al [3] describe the high incidence of reduced factor (F)XIII levels in their prospective observational study and its association with bleeding during ECMO. Previously, little has been described about this key component of stable clot formation in this setting. In a cohort of 101 patients, 73% had FXIII deficiency (FXIIID), defined as a level <70%, with 42% of these deficient patients having a bleeding episode during the ECMO run in comparison with 11% those without. Previous studies have described similar high prevalence of FXIIID during ECMO in 67% to 93% of patients [4,5]. This study provides a larger patient cohort with consistent longitudinal testing and confirms these smaller retrospective studies. They noted that FXIIID was not associated with a difference in time to first bleeding event. The authors also describe further granularity on these patients with a higher incidence of FXIIID in patients with viral and SARS-CoV-19 (COVID-19) infection and longer ECMO runs with venovenous circuits.

Acquired FXIIID is distinct from other acquired coagulation defects that are typically driven by autoimmunity and malignancy. Acquired FXIIID has been associated to trauma, large-volume blood transfusion and surgery, and its presence is associated with poorer clinical outcomes, including mortality and wound healing [[6], [7], [8], [9]]. Of note, a larger decrease in FXIII levels in critically unwell patients with COVID-19 infection has been described to have a higher risk of mortality, reflecting a potential associated variable seen in this ECMO cohort and a disease with distinct coagulopathy during ECMO [6]. It is of interest that most of these patients had FXIIID at ECMO initiation and subsequent longitudinal samples remained similar, showing that once FXIIID develops, it persists throughout the run. There was a modest, nonsignificant increase in FXIII levels following decannulation. These findings suggest a potential multifactorial effect of the underlying disease, critical illness, hemodilution, and ECMO-surface adherence to the development of FXIIID.

Factor XIII is a heterotetramer composed of 2 A and 2 B subunits. The function of FXIII is to crosslink extensive fibrin multimers by their α- and γ-domains to form a stable, strong clot structure. FXIIID has a resulting reduction in clot firmness and increases bleeding risk with a decreased resistance to plasmin-mediated fibrinolysis [10,11]. To compound this, both hypofibrinogenemia and increased fibrinolytic activity are seen during ECMO, further leading to reduced clot strength [5,12,13]. Although not well described in ECMO, reduced clot firmness is recognized during cardiopulmonary bypass, with a correlation to FXIII levels [14].

With the recognition that acquired FXIIID may be important in identifying patients at risk of bleeding during ECMO, the questions remain: how to assess for this, when to treat and what treatment options are available (Figure. When assessing coagulation during ECMO, guidelines recommend the use of routine coagulation monitoring, typically platelet counts, prothrombin and activation partial thromboplastin times, and fibrinogen levels, in stable patients to trigger coagulation factor replacement or transfusion beyond a laboratory threshold. These thresholds are usually lessened in those with active bleeding and require rapid turn-around in this setting. Clinical guidelines at present do not suggest routine monitoring of FXIII during ECMO. In the study described by Kompehl et al. [3], there is no suggested significant threshold for bleeding risk, and a level of <70% was used based upon normal reference ranges. This is in comparison with patients with congenital FXIIID who often do not require FXIII replacement until much lower levels of typically <15% [15]. Indeed, the lowest tertile in this cohort of 15% to 49% would still be much higher than this threshold, albeit that the coagulopathy during ECMO has multiple other defects, suggesting a potential higher threshold for FXIII replacement in this scenario.

**Figure:**
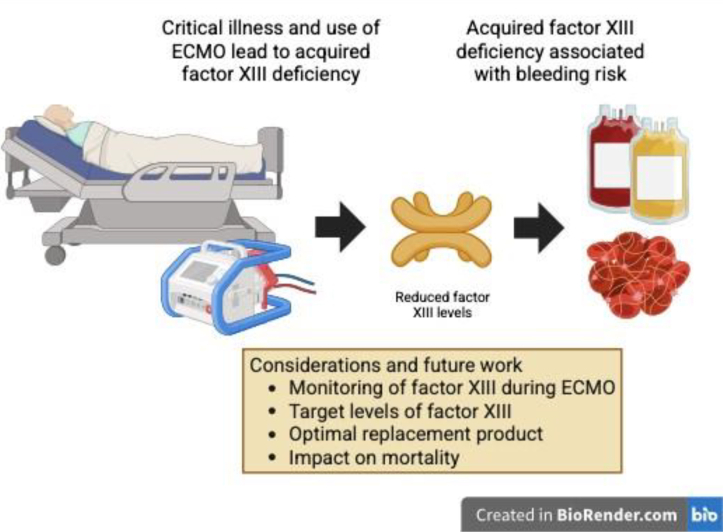
Considerations and impact of factor XIII deficiency during extracorporeal membrane oxygenation (ECMO).

For FXIII testing, there are multiple methods available including enzyme-linked immunoassay, chromogenic assays, and functional methods, such as the ammonia release assay [16]. This study measured FXIII activity levels using a widely used chromogenic platform. Factor XIII assays are not typically performed in real time, and some, particularly functional assays, take significant expertise. Therefore, the applicability of incorporating these into routine clinical practice may be difficult to provide to the clinician at the bedside in a timely fashion. Viscoelastic testing (VET) has been considered in ECMO as a point-of-care test for coagulation monitoring. Nevertheless, its routine use has yet to be established. FXIIID has been demonstrated to reduce various parameters of VET in particular clot strength and increased fibrinolysis [17]. Correlation is worth exploring for detecting FXIIID during ECMO using VET, particularly in patients with active bleeding and normal fibrinogen levels.

Specific FXIII replacement has been described in a limited number of ECMO protocols [5]. Factor XIII can be found in cryoprecipitate, fibrinogen concentrates, and fresh frozen plasma in varying quantities, as well as in commercially available recombinant and plasma-derived FXIII concentrates [15,18,19]. Since many patients on ECMO receive fibrinogen replacement products when they experience bleeding, their FXIII, FVIII, and von Willebrand factor levels may also be unintentionally repleted. A clinical question would be whether the addition of routine FXIII monitoring and specific factor replacement in nonbleeding patients would be of benefit to reduce bleeding risk and its severity further and whether this offsets issues such as increased testing requirement, transfusion burden and exposure to transfusion-related complications. A recent observational study by Fujita et al. [20] showed that in 20 cases of pediatric ECMO, routine FXIII replacement at levels of <30% in nonbleeding or <30% to 50% in bleeding patients decreased the rate of bleeding and its severity in 85% of patients [20].

Although this study provides a potential new target for improving bleeding outcomes with ECMO, there are limitations. First, the authors failed to show an impact upon mortality despite the high incidence of FXIIID. They identified that the majority of bleeds using Bleeding Academic Research Consortium classification were grade ≥2, which have been shown in other populations to have a significant mortality impact upon 28-day survival [21]. Considerations may be that this study was either underpowered to assess the impact upon mortality or the threshold for FXIIID needs to be further defined. Additionally, when multivariate analysis was performed, FXIIID no longer showed an impact on bleeding risk. Furthermore, no FXIII-specific replacement was given in this study to assess its impact upon bleeding. Therefore, previous studies using FXIII in the perioperative setting with high bleeding risk have failed to show improved clinical outcomes [22].

Moving forward, additional research into FXIII and clot stability during ECMO is necessary to identify new ways to reduce stubbornly high bleeding rates, even after recent improvements in anticoagulation and transfusion protocols. Consideration is needed as how best to adopt monitoring of FXIII levels into routine practice, providing threshold recommendations for treatment, and which factor replacement approaches may achieve the best outcomes. This can be performed either solely for FXIII or as part of a broader monitoring approach that incorporates additional coagulation parameters, such as von Willebrand factor assays and VET, beyond those currently considered in current practice.

In summary, Kornfehl et al. [3] describe FXIIID as being common throughout the duration of ECMO and its association with increased bleeding risk. The next challenge will be to assess how these findings can be integrated into monitoring and treatment strategies to lower bleeding risk and reduce mortality.
